# Evaluating the Anti-Inflammatory and Chondroprotective Effects of *Adenocaulon himalaicum* Extract Through Network Pharmacology and Experimental Validation

**DOI:** 10.3390/ijms26030877

**Published:** 2025-01-21

**Authors:** Yun Mi Lee, Eunjung Son, Dong-Seon Kim, Kyu-Suk Shim, Su Hyun Yu

**Affiliations:** 1 KM Science Research Division, Korea Institute of Oriental Medicine, Daejeon 34054, Republic of Korea; 2 Univera Co., Ltd., Cheonan 31257, Republic of Korea

**Keywords:** osteoarthritis, inflammation, *Adenocaulon himalaicum*, chondrocyte protection

## Abstract

Conventional osteoarthritis treatments have several side effects and poor efficacy. This study explored the anti-inflammatory and cartilage-protective effects of *Adenocaulon himalaicum*, with a focus on its potential application in osteoarthritis treatment. The anti-inflammatory effects of *A. himalaicum* extract (AHLE) were investigated in lipopolysaccharide-induced RAW264.7 macrophages, interleukin (IL)-1β-stimulated chondrocytes, and rats with carrageenan-induced hind paw oedema. We also evaluated AHLE’s analgesic activity in mice with acetic acid-induced writhing. The components of AHLE were subjected to network pharmacological analysis to elucidate their mechanisms of action and validate potential pathways and targets in vitro. AHLE markedly reduced nitric oxide, IL-1β, IL-6, tumour necrosis factor-alpha, and prostaglandin E2 production in both RAW264.7 macrophages and chondrocytes. In animal models, AHLE reduced carrageenan-induced hind paw swelling and provided analgesic effects in writhing tests. The main components were chlorogenic acid; 1,3-dicaffeoylquinic acid; 3,4-dicaffeoylquinic acid; 3,5-dicaffeoylquinic acid; and 4,5-dicaffeoylquinic acid. According to network pharmacological analysis, AHLE’s main therapeutic targets are the mitogen-activated protein kinase (MAPK) signalling pathway and extracellular matrix (ECM) degradation. These targets were verified through the MAPK pathway and expression of matrix metalloproteinase, an enzyme involved in ECM degradation. In conclusion, AHLE has considerable anti-inflammatory and cartilage-protective properties, making it a promising candidate for osteoarthritis therapy.

## 1. Introduction

Inflammation is a natural immune response characterized by redness, heat, swelling, pain, and loss of function. It protects the body against various pathogens and environmental dangers [1]. It also plays a significant role in the pathogenesis of various chronic diseases, particularly the development and progression of osteoarthritis (OA) [2]. Furthermore, it mediates the development of pain in patients with OA [3]. Joint pain is a typical symptom of OA that affects physical function and psychological parameters, consequently affecting patient quality of life [4]. Therefore, identifying early inflammatory events and providing timely treatment are important, not only for pain relief but also for preserving joint function and preventing inflammation-induced joint destruction [5].

The anti-inflammatory properties of the perennial plant *Adenocaulon himalaicum* Edgew. have been investigated in previous studies. This plant is native to Korea, Japan, and China and is traditionally consumed as a boiled vegetable or as a soup ingredient [6]. Various parts of this plant, including the leaves, seeds, roots, and stems, have been used as ingredients in Korean folk medicine for treating abscesses, bleeding, and inflammation [7]. Furthermore, *A. himalaicum* has demonstrated excellent antioxidant [6,7], skin damage protection [6], immunostimulatory, anti-obesity [8], and anticancer activities [7,9]. It has been used in the treatment of inflammation, but its anti-inflammatory mechanism and efficacy against OA, an inflammation-related disease, have not been reported.

The debilitating symptoms of OA necessitate the identification of natural products containing bioactive compounds with anti-inflammatory properties to develop alternative strategies for delaying or treating OA. Therefore, in this study, we investigated the main components of *A. himalaicum* and the mechanisms underlying OA treatment through network pharmacological analysis. Subsequently, we aimed to provide important evidence for the therapeutic value of *A. himalaicum* extract (AHLE) in treating OA by mitigating inflammation and preserving cartilage through in vivo and in vitro studies. The findings from macrophages and chondrocytes cell culture experiments confirmed the primary therapeutic targets identified in the network pharmacology analysis, i.e., the MAPK signalling pathway and ECM degradation.

## 2. Results

### 2.1. Quantification of Marker Compounds of AHLE

**Figure 1:**
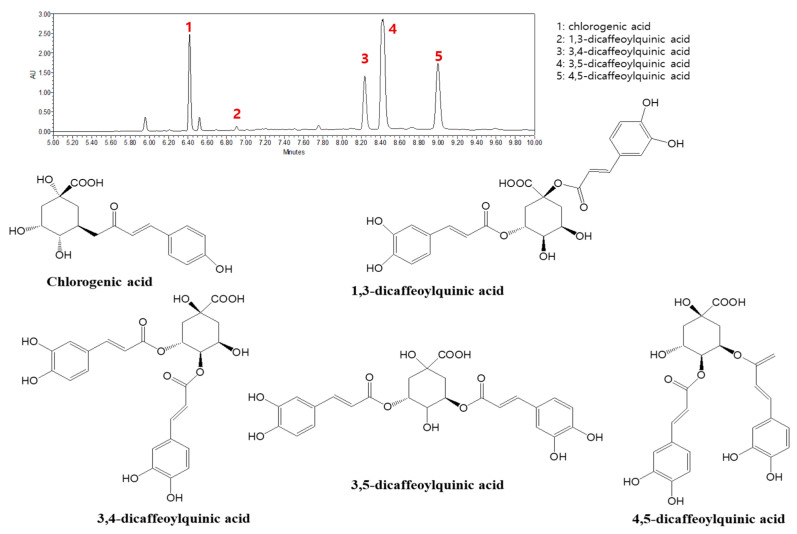
Representative ultrahigh-performance chromatograms (UPLCs) of *A. himalaicum* at 320 nm: (1) chlorogenic acid; (2) 1,3-dicaffeoylquinic acid; (3) 3,4-dicaffeoylquinic acid; (4) 3,5-dicaffeoylquinic acid; and (5) 4,5-dicaffeoylquinic acid.

The major components of AHLE were identified using ultrahigh-performance chromatograms (UPLCs). A comparison of the retention time and mass spectra with those of reference standards revealed five phytochemicals predominantly present in the extract. The quantities of these compounds were 15.4 ± 0.02, 0.5 ± 0.03, 8.1 ± 0.05, 23.6 ± 0.21, and 16.6 ± 0.11 mg/g of chlorogenic acid; 1,3-dicaffeoylquinic acid; 3,4-dicaffeoylquinic acid; 3,5-dicaffeoylquinic acid; and 4,5-dicaffeoylquinic acid, respectively (Figure 1).

### 2.2. Chemical Profiling of A. himalaicum Using UPLC–Tandem Mass Spectrometry (MS/MS)

**Figure 2:**
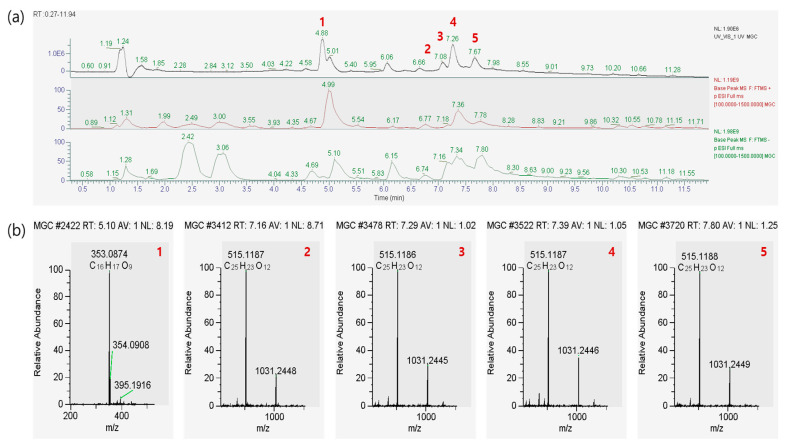
Representative total ion chromatograms of *A. himalaicum* (**a**) and the standard solution (**b**), as obtained using UPLC−MS/MS: (1) chlorogenic acid; (2) 1,3-dicaffeoylquinic acid; (3) 3,4-dicaffeoylquinic acid; (4) 3,5-dicaffeoylquinic acid; and (5) 4,5-dicaffeoylquinic acid.

Five AHLE components, namely chlorogenic acid; 1,3-dicaffeoylquinic acid; 3,4-dicaffeoylquinic acid; 3,5-dicaffeoylquinic acid; and 4,5-dicaffeoylquinic acid, were measured and detected. Their molecular weights (353.0881, 353.0883, 515.1198, 515.1198, and 415.1198 *m*/*z*, respectively) were detected in the negative mode (Figure 2).

### 2.3. Network Pharmacology-Based Potential Targets of Marker Molecules

**Figure 3:**
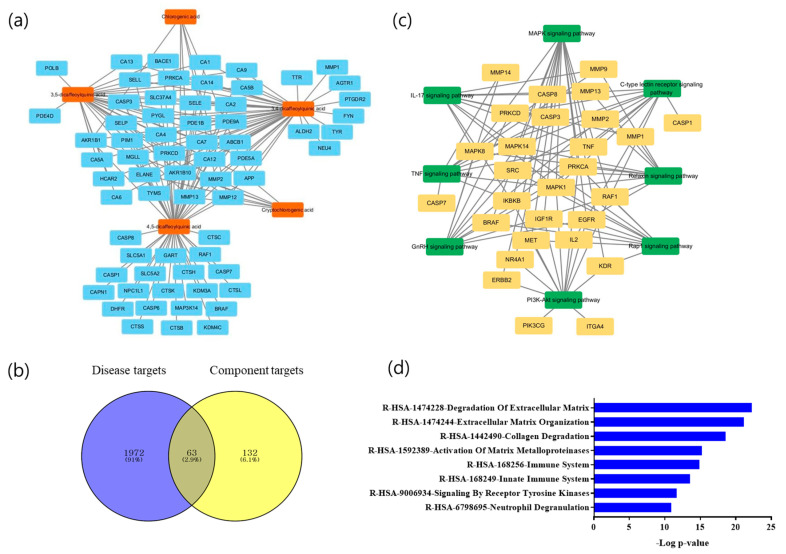
Active ingredients of *Adenocaulon himalaicum* extract (AHLE) and its therapeutic targets for osteoarthritis (OA). (**a**) Network of five molecules and potential target genes; (**b**) Venn diagram of component target–disease target; (**c**) target gene–KEGG pathway network of AHLE; and (**d**) REACTOME pathway analysis.

In total, 195 genes associated with the five molecules were obtained from the SwissTargetPrediction database. In contrast, the GeneCards database revealed 2035 genes associated with OA, chondrocyte protection, and inflammation. Of the targets obtained from SwissTargetPrediction and GeneCards, 63 common targets were identified. We conducted an additional analysis of Kyoto Encyclopedia of Genes and Genomes (KEGG) pathways to explore the mechanisms through which AHLE affects OA. Eight pathways were selected as OA-related pathways based on *p*-values: MAPK, phosphatidylinositol 3′-kinase-Akt, C-type lectin receptor, relaxin, gonadotropin-releasing hormone, interleukin (IL)-17, tumour necrosis factor (TNF), and Ras-proximate-1 signalling pathways (Figure 3c). In the REACTOME pathways (Figure 3d), target genes were enriched in ECM degradation (*p*-value = 5.70 × 10^−23^), ECM organization (*p*-value = 7.21 × 10^−22^), collagen degradation (*p*-value = 2.59 × 10^−19^), and the activation of matrix metalloproteinases (MMPs; *p*-value = 6.11 × 10^−16^). Furthermore, network pharmacology revealed that AHLE regulates inflammation through the MAPK signalling pathway and is involved in ECM destruction, thereby exerting its effects on OA.

### 2.4. Anti-Inflammatory and Analgesic Effects of AHLE In Vivo

**Figure 4:**
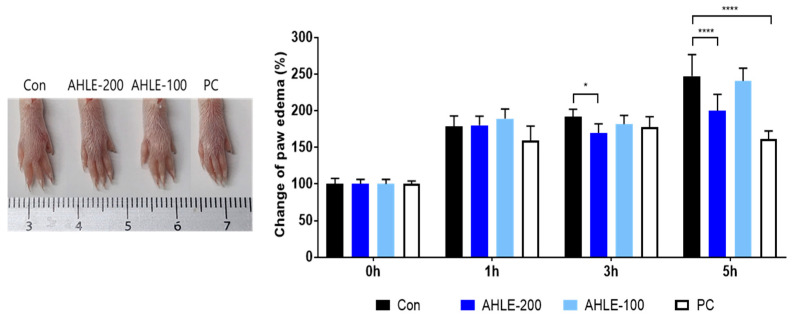
Anti-inflammatory effects of AHLE on carrageenan-induced hind paw oedema in mice. * *p* < 0.05 and **** *p* < 0.0001 compared with control (Con) group. PC, positive control.

We used a carrageenan-induced hind paw oedema mouse model to investigate the anti-inflammatory effects of AHLE. Inflammation was quantified by measuring the volume (mL) of water displaced by the paw in a plethysmometer (Table 1). As shown in Figure 4, oedema reached the maximum at 5 h after the carrageenan injection, whereas 200 mg/kg AHLE and diclofenac, i.e., the positive control (PC), exhibited inhibitory actions of 32.0% and 58.4%, respectively.

We investigated anti-inflammatory mediators including IL-1β, IL-6, prostaglandin E2 (PGE2), and COX-2 in the paws of mice with carrageenan-induced oedema (Table 2). Treatment with 200 mg/kg AHLE significantly reduced the IL-1β, IL-6, PGE2, and COX-2 levels compared to control treatment, with similar results observed for the PC, diclofenac.

**Figure 5:**
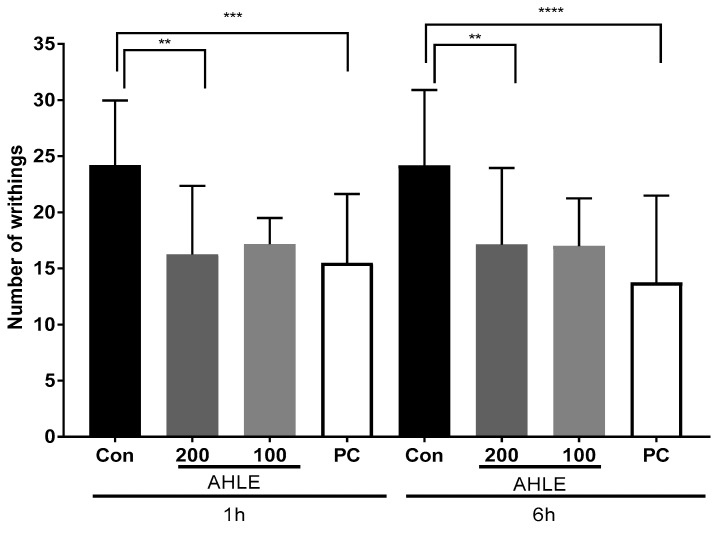
Analgesic effects of AHLE in mice. Reduction in writhing response in mice after treatment with PC and AHLE. ** *p* < 0.01, *** *p* < 0.001, and **** *p* < 0.0001 compared with control (Con) group.

The analgesic effects of AHLE were evaluated based on the writhing response of acetic acid-administered mice. Pain was induced using acetic acid 1 and 6 h after the oral administration of AHLE, and the number of writhing responses was observed. As shown in Figure 5, one hour after drug administration, the reduction in writhing response was similar between the PC and AHLE (200 mg/kg) groups, with reductions of 35.9% and 32.8%, respectively. However, the analgesic effects at 6 h were evaluated as 43.1% and 29.7% in the PC and AHLE (200 mg/kg) groups, respectively, indicating that the effects in the PC group increased over time, whereas those in the AHLE (200 mg/kg) group were similar at both time points. However, the effects observed in the AHLE (200 mg/kg) group were significant at both 1 and 6 h, demonstrating the analgesic effects of this extract.

### 2.5. Regulatory Effects of AHLE on Secretion of Inflammatory Mediators and Cytokines In Vitro

**Figure 6:**
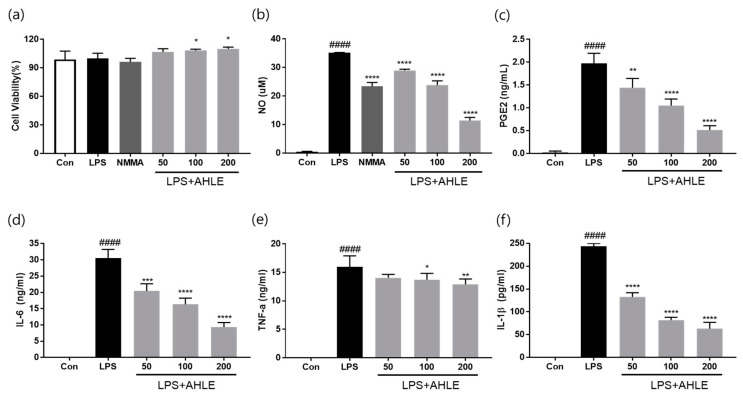
Effects of AHLE on (**a**) cell viability as well as (**b**) nitric oxide (NO), (**c**) prostaglandin E2 (PGE2), (**d**) interleukin-6 (IL-6), (**e**) tumour necrosis factor-α (TNF-α), and (**f**) interleukin-1β (IL-1β) levels in macrophages. #### *p* < 0.0001 compared with control (Con) group. (n = 3) * *p* < 0.05, ** *p* < 0.01, *** *p* < 0.001, and **** *p* < 0.0001 compared with lipopolysaccharide (LPS)-only group.

As shown in Figure 6a, the viability of cells treated with 50, 100, or 200 µg/mL AHLE increased by 6.7%, 8.4%, and 9.7%, respectively (Figure S1A). Lipopolysaccharide (LPS) stimulation markedly increased the levels of inflammatory mediators and cytokines in RAW264.7 cells, whereas AHLE treatment significantly decreased nitric oxide (NO), PGE2, IL-6, TNF-α, and IL-1β secretion (Figure 6b–f). These results confirm that AHLE exhibits anti-inflammatory effects in RAW264.7 cells.

### 2.6. The Effects of AHLE on the Activation of the MAPK Pathway

**Figure 7:**
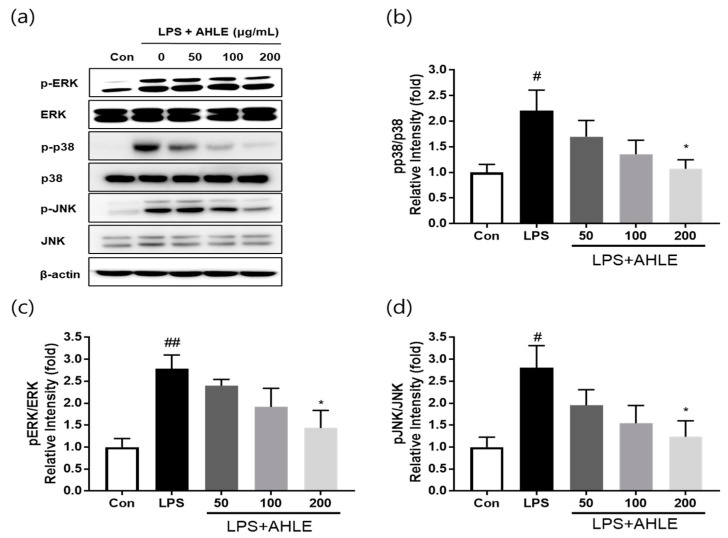
Effects of AHLE on phosphorylation of p-p38, JNK, and ERK in LPS-induced RAW264.7 cells. Western blotting (**a**) and relevant quantitative analyses of (**b**–**d**) p-p38, p38, p-ERK, ERK, p-JNK, and JNK. # *p* < 0.05, and ## *p* < 0.01 compared with control (Con) group. * *p* < 0.05 compared with LPS-only group.

We investigated the phosphorylation levels of p38, ERK, and JNK in LPS-stimulated RAW264.7 cells to determine whether the anti-inflammatory mechanism of AHLE involves the MAPK signalling pathway. LPS stimulation markedly increased p38, ERK, and JNK phosphorylation, whereas AHLE pretreatment reduced it. Overall, this extract reduced p38, ERK, and JNK phosphorylation in a dose-dependent manner (Figure 7).

### 2.7. Effects of AHLE on Inflammation in Chondrocytes

**Figure 8:**
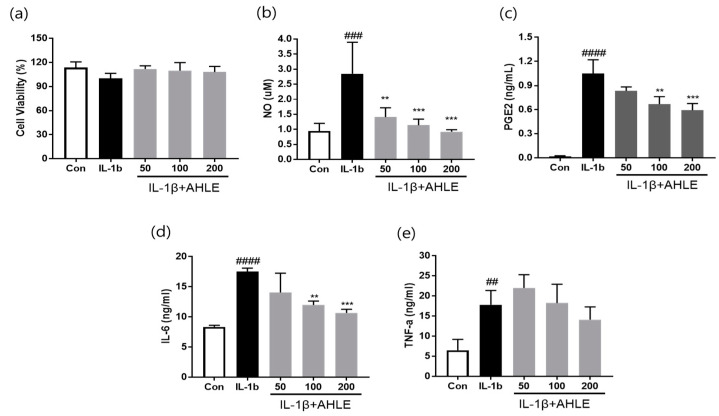
Effects of AHLE on (**a**) cell viability as well as (**b**) NO, (**c**) PGE2, (**d**) IL-6, and (**e**) TNF-α levels in rat primary chondrocytes. ## *p* < 0.01, ### *p* < 0.001, and #### *p* < 0.0001 compared with control (Con) group. (n = 3) ** *p* < 0.01, and *** *p* < 0.001 compared with IL-1β-only group.

The anti-inflammatory effects of AHLE on rat primary chondrocytes were further analysed. Cells were treated with 50, 100, or 200 µg/mL of the extract; all concentrations had no effect on cell proliferation (Figure S1B). Furthermore, IL-1β stimulation significantly increased the expression levels of inflammatory mediators and cytokines in rat primary chondrocytes. However, AHLE treatment considerably reduced the secretion of NO, PGE2, IL-6, TNF-α, and IL-1β (Figure 8). These results confirm that AHLE exhibits anti-inflammatory effects in both RAW264.7 cells and rat primary chondrocytes.

### 2.8. Cytokine/Chemokine Array Analysis

**Figure 9:**
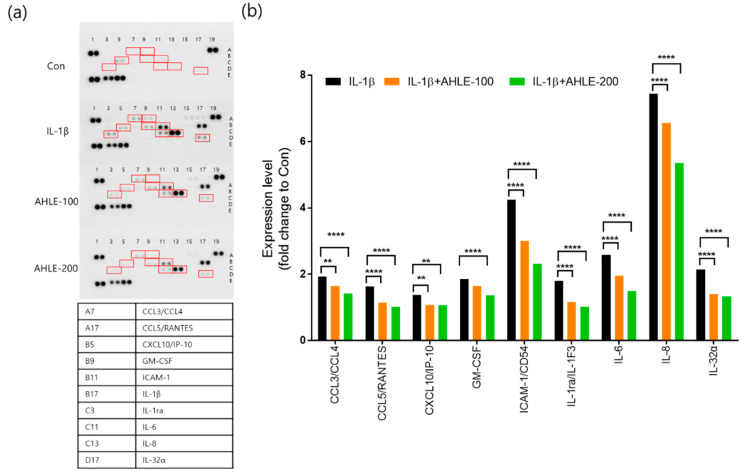
Levels of inflammatory cytokines in SW1353 cells. Cytokine array profiling and numerical values of dot blots representing cytokine protein levels (**a**). Quantification of results of dot blot duplicates from panel (**b**). ** *p* < 0.01, and **** *p* < 0.0001 compared with IL-1β-treated group.

We evaluated the levels of 36 cytokines and chemokines in conditioned media using a human cytokine array membrane to investigate the effects of AHLE on the production of inflammatory cytokines in pooled SW1353 cells. The expression levels of various molecules were quantified as the relative expression values of the control group treated with IL-1β and the group pretreated with AHLE relative to the untreated control (Con) in SW1353 cells. The most significant changes in the production of various molecules induced by IL-1β exposure are shown in Figure 9a. Pretreatment with AHLE reduced the secretion of various chemokines and cytokines (CCL3/CCL4, CCL5, CXCL10, GM-CSF, ICAM-1, IL-1ra, IL-6, IL-8, and IL-32α), as illustrated in Figure 9b.

### 2.9. Effects of AHLE on MMP Expression

We evaluated MMP expression levels in IL-1β-induced SW1353 cells using an enzyme-linked immunosorbent assay (ELISA) and quantitative reverse transcriptase polymerase chain reaction analysis to determine whether they were affected by pretreatment with AHLE.

**Figure 10:**
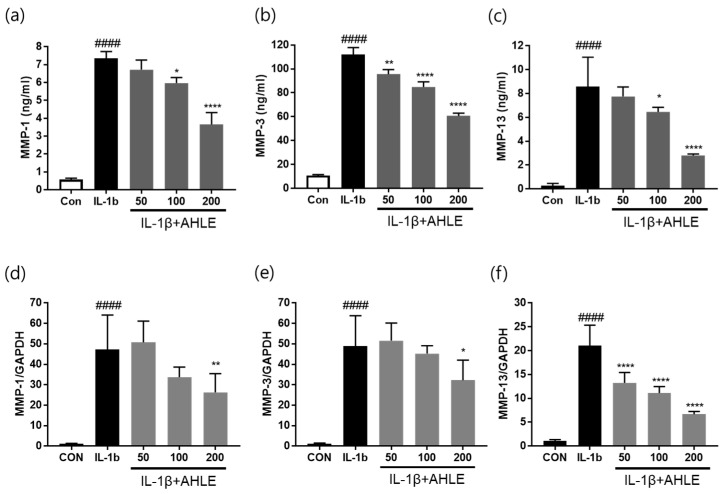
The effects of AHLE on the expression of MMP-1, MMP-3, and MMP-13 in SW1353 cells. The levels of MMP-1 (**a**), MMP-3 (**b**), and MMP-13 (**c**) production, as determined using ELISA kits; the quantitative reverse transcriptase polymerase chain reaction analysis of *MMP1* (**d**), *MMP3* (**e**), and *MMP13* (**f**) expression in SW1353 cells treated with IL-1β. #### *p* < 0.0001 compared with the control (Con) group. * *p* < 0.05, ** *p* < 0.01, and **** *p* < 0.0001 compared with the IL-1β-only group.

The protein and gene expression levels of MMP1, MMP3, and MMP13 were remarkably elevated following treatment with IL-1β. However, AHLE treatment significantly decreased the expression of these MMPs in a concentration-dependent manner. In addition, pretreatment with 200 μg/mL AHLE significantly inhibited the increase in MMP-1, MMP-3, and MMP-13 mRNA expression levels compared to IL-1β treatment alone (Figure 10). This suggests that AHLE regulates the synthesis of catabolic factors (MMP-1, MMP-3, and MMP-13), consequently exerting anticatabolic effects in SW1353 cells exposed to IL-1β.

## 3. Discussion

OA is a chronic degenerative disease that damages joint cartilage, resulting in inflammation, pain, and functional impairment [10]. The incidence of OA is increasing, owing to the aging population. However, no effective treatment has been developed to date. In addition, the drugs currently available cause numerous side effects, including gastrointestinal discomfort and kidney damage [11,12]. OA treatment seeks to reduce inflammation in the joints and prevent cartilage damage to relieve pain. Thus, extensive research has been conducted on the use of herbal medicine as an adjuvant therapy [10,13,14].

To validate the anti-inflammatory mechanism of *A. himalaicum* and to confirm the symptom-relieving effects of OA, the present study employed network pharmacology to identify the key therapeutic targets of *A. himalaicum.* Network pharmacology is a method that applies the pharmacological mechanism of a drug to a biological network to reveal the relationship between components, targets, and diseases [15]. In this study, we used network pharmacology to identify the potential targets and pathways affected by AHLE components including chlorogenic acid; 1,3-dicaffeoylquinic acid; 3,4-dicaffeoylquinic acid; 3,5-dicaffeoylquinic acid; and 4,5-dicaffeoylquinic acid during OA treatment. KEGG analysis revealed that AHLE acts mainly through the MAPK signalling pathway to treat OA. In addition, REACTOME analysis indicated that the molecular mechanism of AHLE involves ECM degradation (Figure 3).

Macrophages play crucial roles in the onset and progression of OA. They regulate inflammatory responses, damage cartilage, and coordinate immune responses [16]. Reducing inflammation is one of the primary goals in the treatment of OA, and suppressing the inflammatory responses of macrophages may alleviate OA symptoms [17]. Resident cells such as macrophages and synoviocytes in the surrounding joint tissues secrete inflammatory cytokines into the synovial fluid. These cytokines stimulate chondrocytes located in the superficial layer of the articular cartilage to produce inflammatory mediators and cartilage-degrading enzymes. Inflammatory mediators and cartilage-degrading enzymes promote the progression of joint inflammation and cartilage degeneration, thereby increasing the severity of OA [18,19]. Therefore, we investigated the anti-inflammatory effects of AHLE on macrophages. RAW 264.7 cells are a mouse-derived macrophage cell line commonly used for inflammation and immunity studies. These cells are typically stimulated with LPS to induce inflammatory responses, consequently promoting the release of inflammatory mediators such as NO, PGE2, and cytokines (e.g., TNF-α, IL-1β, and IL-6) [20]. Our results showed that AHLE alleviated the inflammatory response by reducing the expression of these mediators and cytokines. Furthermore, we investigated the anti-inflammatory effects of AHLE on chondrocytes and the expression of cartilage-degrading enzymes, specifically MMPs, in vitro. The SW1353 chondrosarcoma cell line is commonly used as an alternative to primary adult articular chondrocytes and serves as an in vitro OA model. However, because SW1353 cells cannot fully replace primary chondrocytes, we used them together with primary chondrocytes from rats [21,22,23]. Consistent with RAW 264.7 cells, primary rat chondrocytes stimulated with IL-1β showed increased expression levels of inflammatory mediators such as NO, PGE2, TNF-α, and IL-6, which were significantly reduced by AHLE (Figure 8). The expression of IL-8, ICAM-1, IL-6, and IL-32a in IL-1β-stimulated SW1353 cells was more than double that in unstimulated cells. AHLE (200 mg/kg) considerably reduced IL-8, ICAM-1, IL-6, and IL-32a expression by 28.1%, 45.4%, 42.3%, and 38.0%, respectively (Figure 9). These results suggest that AHLE suppresses the expression of IL-1β, TNF-α, and IL-6, which are the most important inflammatory mediators in the pathogenesis of OA. In addition, this extract regulates various chemokines (IL-8, CCL5, CCL3, and CCL4) that affect the aggravation of the inflammatory state of joints, thereby demonstrating an anti-inflammatory effect [24]. IL-1β, a pro-inflammatory factor, can drive the release of cartilage-degrading enzymes, particularly MMPs. Consistent with these findings, MMP production was elevated in IL-1β-treated SW1353 cells and chondrocytes in the present study (Figure 10 and Figure S2). However, AHLE inhibited MMP production in SW1353 cells and chondrocytes stimulated with IL-1β, suggesting that it may confer a cartilage-protective effect by alleviating ECM degradation.

In the present study, the predicted MAPK signalling pathway was verified using Western blotting in LPS-treated RAW 264.7 cells. These results indicate that AHLE can significantly inhibit the phosphorylation of p38, ERK, and JNK in the MAPK pathway. The activation of the MAPK pathway leads to inflammation and ECM degradation in OA. MAPK pathways (ERK, JNK, p38) can be activated by various inflammatory stimuli such as cytokines (e.g., TNF-α and IL-1β), which in turn increases MMP production, thereby increasing ECM degradation. ECM degradation can further perpetuate inflammation by releasing ECM-derived mediators that recruit and promote immune cell activation. Therefore, by inhibiting MAPK, tissue damage can be alleviated by reducing inflammatory cytokine production and ECM degradation [25,26,27].

Carrageenan-induced paw oedema animal models are commonly used to assess anti-inflammatory activity and are characterized by swelling and pain over time [28,29]. Paw oedema was induced using carrageenan, and the swelling was more than twice that of the control group, as measured using a plethysmometer after 3 h. Compared to the control group, AHLE treatment (200 mg/kg) significantly inhibited paw oedema at 3–5 h after carrageenan injection. Furthermore, AHLE and PC showed anti-inflammatory activity indicated by a significant decrease in the pro-inflammatory cytokines IL-1β, IL-6, PGE2, and COX-2. An acetic acid-induced writhing test, a model commonly used to screen new agents for peripheral analgesic and anti-inflammatory properties, was also conducted in the present study [30,31]. AHLE demonstrated significant analgesic effects within 1 and 6 h of treatment. These results demonstrate the anti-inflammatory and anti-nociceptive effects of AHLE in vivo, thus indicating its potential use as a therapeutic agent for relieving inflammation and inflammatory pain.

AHLE contains multiple ingredients, including chlorogenic acid [32]; 1,3-dicaffeoylquinic acid [33]; 3,5-dicaffeoylquinic acid [34]; and 4,5-dicaffeoylquinic acid [35], which are the main components of AHLE. These components have anti-inflammatory and cartilage-protective effects on macrophages and chondrocytes, respectively. However, the anti-inflammatory and chondroprotective effects of 3,4-dicaffeoylquinic acid have not been reported thus far; therefore, further studies are needed to determine whether 3,4-dicaffeoylquinic acid is the active component in AHLE or whether multiple compounds exert synergistic effects. Future research must determine whether AHLE can be used as an alternative treatment for OA using animal models that closely mimic this disease.

## 4. Materials and Methods

### 4.1. Preparation of Extracts

*Adenocaulon himalaicum* Edgew. leaves were collected from the Obong Mountain (Jeonbuk, Republic of Korea) in September 2021 and identified by Professor Geung-Joo Lee (Chungnam National University, Republic of Korea). A voucher specimen was deposited in the Korean Herbarium of Standard Herbal Resources of the Korea Institute of Oriental Medicine (KIOM201401009503, Dajeon, Republic of Korea). Dried *A. himalaicum* leaves (100 g) were finely powdered and extracted with 1.5 L of 65% ethanol for 3 h at reflux. AHLE was concentrated under reduced pressure, freeze-dried, and stored at 4 °C.

### 4.2. Chemical Profiling Using UPLC and UPLC-MS/MS

The major components of AHLE were quantified using reference standards. A Waters Acquity UPLC system (Waters, Milford, MA, USA) equipped with a quaternary pump, auto-sampler, and photodiode array detector with XBridge^TM^ C18 (4.6 × 250 mm, 5 μm) was used. Gradient elution was performed with solvent A (0.1% phosphoric acid in water) and solvent B (acetonitrile) at a flow rate of 1.0 mL/min as follows: 0–5 min, 5–5% B; 5–10 min, 5–15% B; 10–12 min, 15–5% B; and 12–14 min, 5–5% B. The detection wavelength was 320 nm. The column temperature was 40 °C, and the injection volume was 10 µL. Phytochemical constituents were analysed using a Dionex UltiMate 3000 system coupled with a Thermo Q Exactive mass spectrometer (Thermo Fisher Scientific, Waltham, MA, USA) [36]. An Acquity BEH C18 column (100 × 2.1 mm, 1.7 μm) with 0.1% formic acid in water and acetonitrile was used, and gradient elution was performed. The Q Exactive mass spectrometer was equipped with a heated electrospray ionization source and operated in positive and negative ion-switching modes. The spectrum was obtained in full MS and data-dependent MS2 scan mode. The full acquisition parameters were as follows: resolution, 70,000; scan range, 100–1500 *m*/*z*; and profile spectrum type. The following MS2 parameters were used: resolution, 17,500; loop count, 10; normalized collision energy, 25; and centroid spectrum type. Data acquisition and analysis were performed using the Thermo Xcalibur v.3.0 and Tracefinder v.3.2 (Thermo Fisher Scientific, Bremen, Germany).

### 4.3. Network Pharmacological Analysis

Based on SwissTargetPrediction with ‘Homo sapiens’ species and probability, small molecules and potential target genes related to the five marker compounds of AHLE were collected. To rank the targets in this database and assess the accuracy of the predictions, we used the probability resulting from our cross-validation analysis. Target genes were identified by searching GeneCards: Human Gene Database according to the intersection of the genes related to active small molecules and OA-related genes. The EnrichR program was used to identify important signalling pathways [37]. For gene set analysis, KEGG and REACTOME gene sets were used with a *p*-value < 0.05. Network visualization was conducted using Cytoscape version 3.7.2 (Cytoscape, Boston, MA, USA).

### 4.4. Cell Culture

The RAW264.7 murine macrophage cell line and human SW1353 cells (American Type Culture Collection, Rockville, MD, USA) were cultured in Dulbecco’s modified Eagle medium (DMEM) or DMEM/F-12 medium supplemented with 10% foetal bovine serum (FBS; Gibco BRL, Grand Island, NY, USA) and penicillin/streptomycin (100 unit/100 µg/mL) in a CO_2_ incubator (37 °C; 5% CO_2_). Primary chondrocytes were obtained from the knee joint cartilage of 4-week-old Sprague–Dawley rats. The articular surface cartilage of the knee joint was isolated, cut into pieces, and incubated in 0.2% collagenase for 24 h at 37 °C. Next, the chondrocytes were filtered using a mesh screen, centrifuged, and cultured in DMEM/F-12 supplemented with 10% FBS under 5% CO_2_ at 37 °C. RAW 264.7 cells were seeded on a 96-well plate at 1 × 10^5^ cells/well. After 24 h, the medium was replaced with serum-free DMEM containing different concentrations of AHLE (50, 100, and 200 µg/mL) with or without LPS (0.5 µg/mL), and the cells were incubated in this medium for an additional 24 h. Next, SW1353 cells and primary chondrocytes were seeded in 96-well plates at 5 × 10^4^ cells/well and cultured for 24 h. Thereafter, media containing different concentrations of AHLE (50, 100, and 200 µg/mL) with or without IL-1β (10 ng/mL) were added to the cells [38], which were then cultured for another 24 h. Control (Con) solution refers to serum-free DMEM without LPS or IL-1β.

### 4.5. Cell Viability

A CCK-8 assay was conducted according to the manufacturer’s instructions to evaluate the cytotoxicity of AHLE. After 24 h of AHLE pretreatment, 10 μL CCK-8 reagent was added to each well. The cells were then incubated for 4 h, and optical density was measured at 450 nm using a microplate reader (BioTek Instruments, Winooski, VT, USA).

### 4.6. Enzyme-Linked Immunosorbent Assay (ELISA)

The cell culture supernatant in each group was collected, and the amounts of secreted cytokines and MMPs were evaluated using the corresponding ELISA kits (Bio-Rad, Hercules, CA, USA).

### 4.7. Microarray and Western Blot Analyses

Total protein from cells was evaluated as described in the Supplementary Materials.

### 4.8. Carrageenan-Induced Hind Paw Oedema

ICR mice were classified into four groups (n = 5 per group) and orally administered AHLE (100 and 200 mg/kg) or diclofenac (10 mg/kg; Sigma-Aldrich, St. Louis, MO, USA) as a PC. After 1 h, each mouse was administered 1% carrageenan suspension (0.05 mL per animal) in the left hind paw. The thickness of the hind paw of each mouse was measured at 0, 1, 3, and 5 h after the carrageenan injection using a plethysmometer (LE 7500; Panlab Harvard Apparatus, Barcelona, Spain).

### 4.9. Writhing Test

ICR mice were classified into eight groups (n = 5 per group) and treated with AHLE (100 and 200 mg/kg) or diclofenac (10 mg/kg; Sigma-Aldrich) as a PC. The mice were orally administered the samples and intraperitoneally injected with 0.75% acetic acid (10 mL/kg) at 1 h and 6 h after sample treatment, respectively. The writhing reaction was observed after 5 min and assessed for 10 min. This experiment was repeated thrice to confirm its effectiveness.

### 4.10. Statistical Analysis

Statistical analysis was performed with GraphPad Prism 7 (GraphPad Software, San Diego, CA, USA). All data are presented as the mean ± standard deviation. An analysis of variance with Dunnett’s multiple comparison test was used to identify statistically significant differences among the groups.

## Figures and Tables

**Table 1:** Carrageenan-induced paw oedema test.

Group	Paw Volume (mL) After Induction (Mean ± SD)			
	0 h	1 h	3 h	5 h
Con	0.13 ± 0.01	0.23 ± 0.02	0.25 ± 0.01	0.33 ± 0.03
AHLE-200	0.13 ± 0.01	0.22 ± 0.02	0.22 ± 0.02 *	0.26 ± 0.03 ****
AHLE-100	0.13 ± 0.01	0.25 ± 0.02	0.24 ± 0.02	0.32 ± 0.02
PC (diclofenac)	0.13 ± 0.01	0.21 ± 0.03	0.24 ± 0.02	0.22 ± 0.02 ****

Con, control; AHLE-200, 200 mg/kg AHLE; AHLE-100, 100 mg/kg AHLE. * *p* < 0.05 and **** *p* < 0.0001 compared with control (Con) group. PC, positive control.

**Table 2:** Effect of AHLE on cytokine expression in carrageenan-induced oedematous paw tissue.

Group	IL-1β (ng/g)	IL-6 (ng/g)	COX-2 (ng/g)	PGE2 (pg/g)
Con	3.73 ± 0.86	0.69 ± 0.09	3.23 ± 0.85	143.56 ± 6.38
AHLE-200	1.59 ± 1.09 *	0.51 ± 0.10 **	1.60 ± 0.33 **	104.81 ± 4.23 ***
AHLE-100	2.88 ± 1.22	0.58 ± 0.04	2.64 ± 0.34	124.52 ± 1.55
PC (diclofenac)	2.75 ± 1.15	0.27 ± 0.06 ****	1.36 ± 1.02 **	88.80 ± 4.96 ****

* *p* < 0.05, ** *p* < 0.01, *** *p* < 0.001, and **** *p* < 0.0001 compared with the control (Con) group. PC, positive control.

## Data Availability

All data are available within this article.

## Supplementary Materials

The following supporting information can be downloaded at https://www.mdpi.com/article/10.3390/ijms26030877/s1.
