# Supplementary material for: Evaluating the Anti-Inflammatory and Chondroprotective Effects of Adenocaulon himalaicum Extract Through Network Pharmacology and Experimental Validation
Source: Int J Mol Sci. 2025 Jan 21;26(3):877. doi: 10.3390/ijms26030877 (PMC11816759; doi:10.3390/ijms26030877)
Supplement: Supplementary file 1 [file ijms-26-00877-s001.zip › ijms-3335991-supplementary.pdf]

(Supplementary method)

Western blotting

Total protein was extracted from the cells using RIPA buffer. Twenty micrograms of protein was separated using 10% SDS-PAGE and subsequently transferred onto PVDF membranes (Millipore, Burlington, MA, USA). The membranes were blocked with 5% non-fat milk in tris-buffered saline containing 0.05% Tween-20 for 1 h and incubated with primary antibodies overnight at 4 °C, followed by HRP-labeled secondary antibodies for 1 h at room temperature. They were then washed with TBST, and signals were detected using an image analyzer (LAS 4000 mini; GE Healthcare Bio-Sciences, NJ, USA) with enhanced chemiluminescence solution. β-actin was used as the internal reference, and relative expression was quantified using ImageJ software (National Institutes of Health, Bethesda, MD, USA).

Microarray analysis

Cytokines in SW1353 cells were quantified using the Proteome Profiler Human Cytokine Array Kit (ARY005B; R&D Systems, Minneapolis, MN, USA) containing 36 cytokines spotted on a nitrocellulose membrane according to the manufacturer's protocol. Protein quantification was performed by analyzing the pixel density of each spot using the ImageJ software.

(Supplementary data)

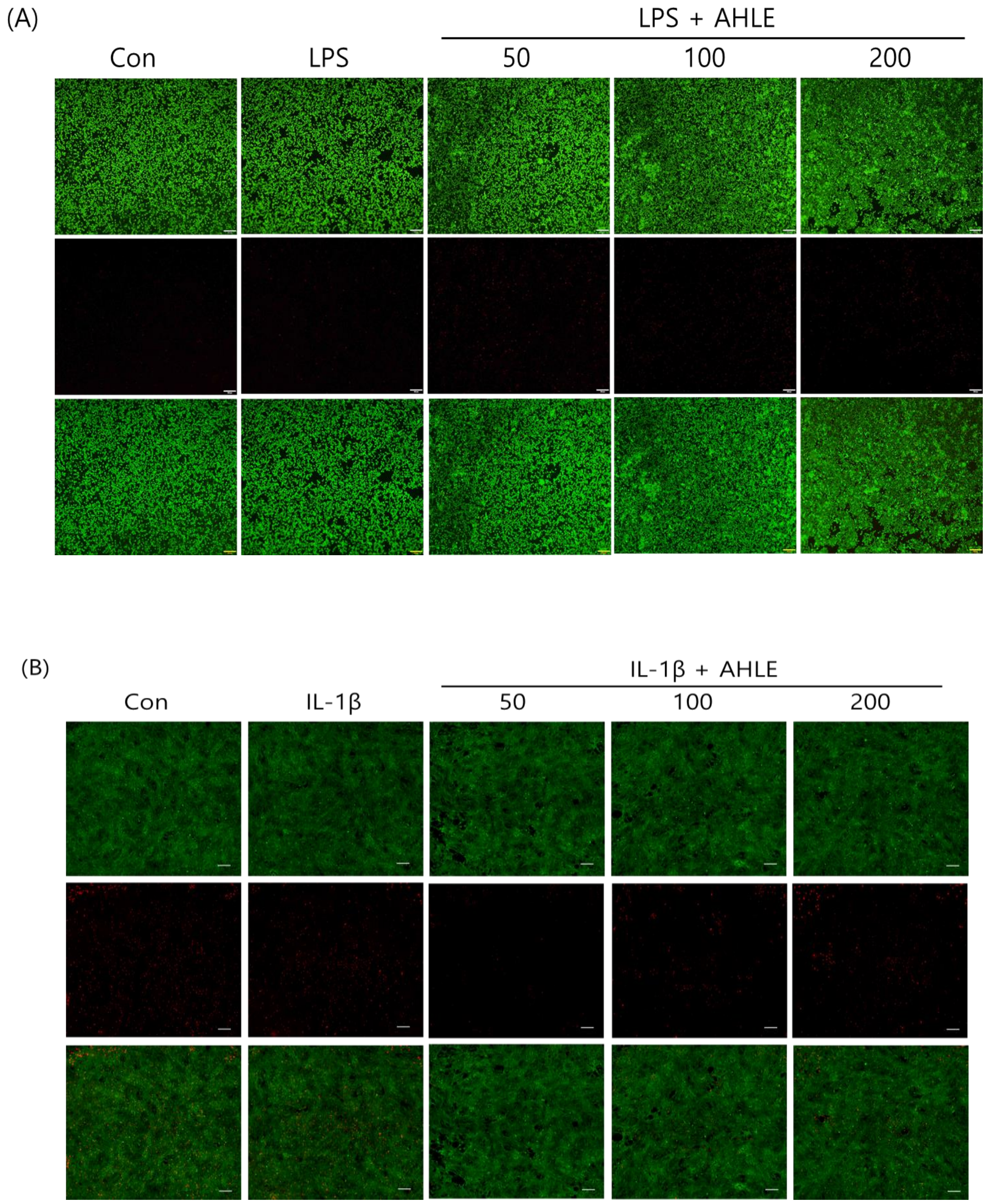

Figure S1. Fluorescence microscopy images of cell viability of RAW264.7 macrophages (A) and SW1353 cell (B). Cells are stained green, and dead cells red. (scale bar; 100 μm).

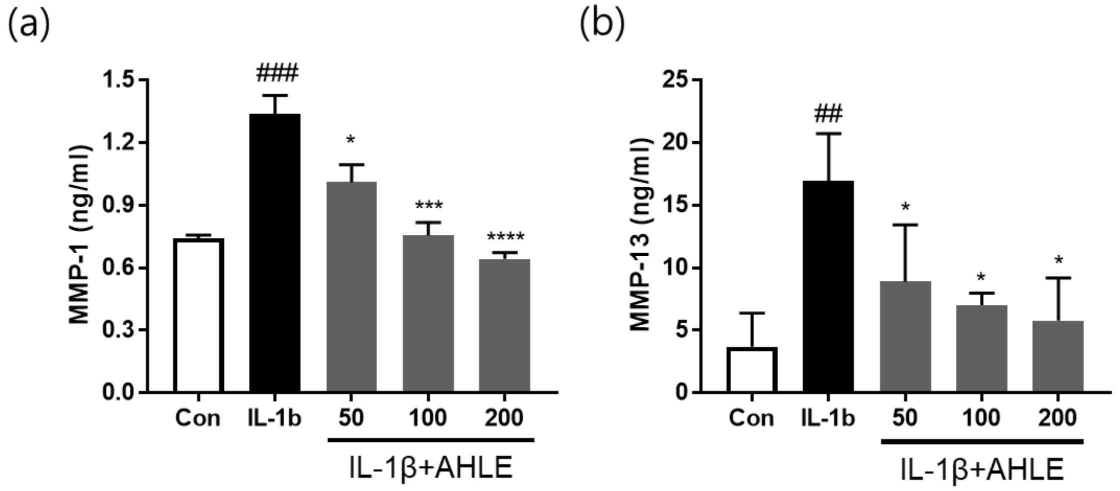

Figure S2. Effects of AHLE on the expression of MMP-1, and MMP-13 in rat primary chondrocytes. The levels of MMP-1 (**a**), and MMP-13 (**b**) production, as determined using ELISA kits. ## $p < 0.01$, and ### $p < 0.001$ compared with the Control (Con) Group. * $p < 0.05$, *** $p < 0.001$, and **** $p < 0.0001$ compared with the IL-1β-only group.
